# Meteorological gaps in audits of pedestrian environments: a scoping review

**DOI:** 10.1186/s12889-024-19441-6

**Published:** 2024-07-27

**Authors:** H. F Drapeau, P. Singh, F. Benyaminov, K. Wright, J. C. Spence, S. Nuzhat, A. Walsh, K. Islam, Z. Azarm, K. K. Lee

**Affiliations:** 1 Housing for Health, Division of Preventive Medicine, Department of Medicine, University of Alberta, Edmonton, AB Canada; 2 Faculty of Kinesiology, Sport, and Recreation, University of Alberta, Edmonton, AB Canada

**Keywords:** Climate change, Weather, Season, Winter, Built environment, Physical activity, Walkability, Active transportation

## Abstract

**Background:**

Weather and season are determinants of physical activity. Therefore, it is important to ensure built environments are designed to mitigate negative impacts of weather and season on pedestrians to prevent these losses. This scoping review aims to identify built environment audits of pedestrian environments developed for use during a specific weather condition or season. Secondly, this review aims to investigate gaps in the inclusion of relevant weather mitigating built environment features in pedestrian environment audit tools.

**Methods:**

Following a standard protocol, a systematic search was executed in CINAHL, Medline and Web of Science to identify built environment audit tools of pedestrian spaces. These databases were chosen since they are well-known to comprehensively cover health as well as multi-disciplinary research publications relevant to health. Studies were screened, and data were extracted from selected documents by two independent reviewers (e.g., psychometric properties and audit items included). Audit items were screened for the inclusion of weather mitigating built environment features, and the tool’s capacity to measure temperature, precipitation, seasonal and sustainability impacts on pedestrians was calculated.

**Results:**

The search returned 2823 documents. After screening and full text review, 27 articles were included. No tool was found that was developed specifically for use during a specific weather condition or season. Additionally, gaps in the inclusion of weather mitigating items were found for all review dimensions (thermal comfort, precipitation, seasonal, and sustainability items). Poorly covered items were: (1) thermal comfort related (arctic entry presence, materials, textures, and colours of buildings, roads, sidewalk and furniture, and green design features); (2) precipitation related (drain presence, ditch presence, hazards, and snow removal features); (3) seasonal features (amenities, pedestrian scale lighting, and winter destinations and aesthetics); and (4) sustainability features (electric vehicle charging stations, renewable energy, car share, and bike share facilities).

**Conclusions:**

Current built environment audit tools do not adequately include weather / season mitigating items. This is a limitation as it is important to investigate if the inclusion of these items in pedestrian spaces can promote physical activity during adverse weather conditions. Because climate change is causing increased extreme weather events, a need exists for the development of a new built environment audit tool that includes relevant weather mitigating features.

**Supplementary Information:**

The online version contains supplementary material available at 10.1186/s12889-024-19441-6.

## Background

 It is well established that environmental conditions, including weather and seasonality, affect physical activity (PA) [1–6]. Temperature and PA have a non-linear relationship, with PA increasing with temperature until approximately 25–29 °C then decreasing again, with dramatic PA decreases above 40 °C [2, 7, 8]. In areas that receive high amounts of solar radiation (e.g., areas with limited shade) there can be further losses in PA during these hot days [9]. Additionally, prolonged precipitation (e.g., snow, rain) can also reduce both leisure PA and active transport [7, 10]. In older adults, snow and ice can be a large barrier to walking/wheeling leading to large decreases in PA during winter [11]. As global temperatures increase and severe weather conditions (e.g., precipitation events, drought) become more frequent [12], the negative impacts of environment on PA could become exacerbated [7]. This is of concern for public health since ~ 1/3 of the global population do not meet PA guidelines [13], contributing to an increased likelihood of death [14], and $67.5 billion in healthcare costs and productivity losses [15].

Though it is not possible to control the weather, and climate change impacts are already underway, outdoor areas can be adapted to protect against weather conditions [16]. Features of urban design have also been found to worsen extreme weather events. For example, the heat island effect is a phenomenon where urban areas become significantly hotter than air temperatures due to insufficient vegetation and construction materials that reflect the heat [17, 18]. To combat these issues and prepare for future climate changes, urban design and planning organizations have proposed and implemented features to modify local micro-climates and create urban spaces that might be cooler, hotter, or drier than surrounding areas [18–21].

Walking, cycling, and wheeling are among the most popular forms of PA [14], and heavily rely on supportive pedestrian streetscapes [4, 22, 23]. As weather and climate conditions change, and to mitigate further climate changes through promotion of active transportation modes like walking, it is increasingly important for urban design and planning to consider impact of weather on pedestrians. To understand the role of urban design features in promoting outdoor PA under various meteorological conditions and to address them adequately in different jurisdictions, it is necessary to measure the presence/extent/quality of these features in pedestrian environments [24]. However, the extent to which measures of the built and/or pedestrian environment address aspects of meteorological conditions is unknown. Recently, a few popular built environment audit tools [25] were scrutinized for their inability to adequately measure winter features of pedestrian built environments [26]. Thus, there is an urgent need for consistent and comprehensive assessment tools for the capacity of pedestrian spaces to mitigate impacts of weather and seasonality on pedestrians and their PA.

This paper presents a comprehensive scoping review to identify audit tools designed for use during specific seasons or weather conditions. Additionally, those tools not specifically designed for season or weather condition, were assessed for their inclusion of built environment features that might mitigate impacts of weather and seasons on pedestrians (e.g., vegetation, building overhangs, winter aesthetics, transit availability).

## Methods

**Fig. 1:**
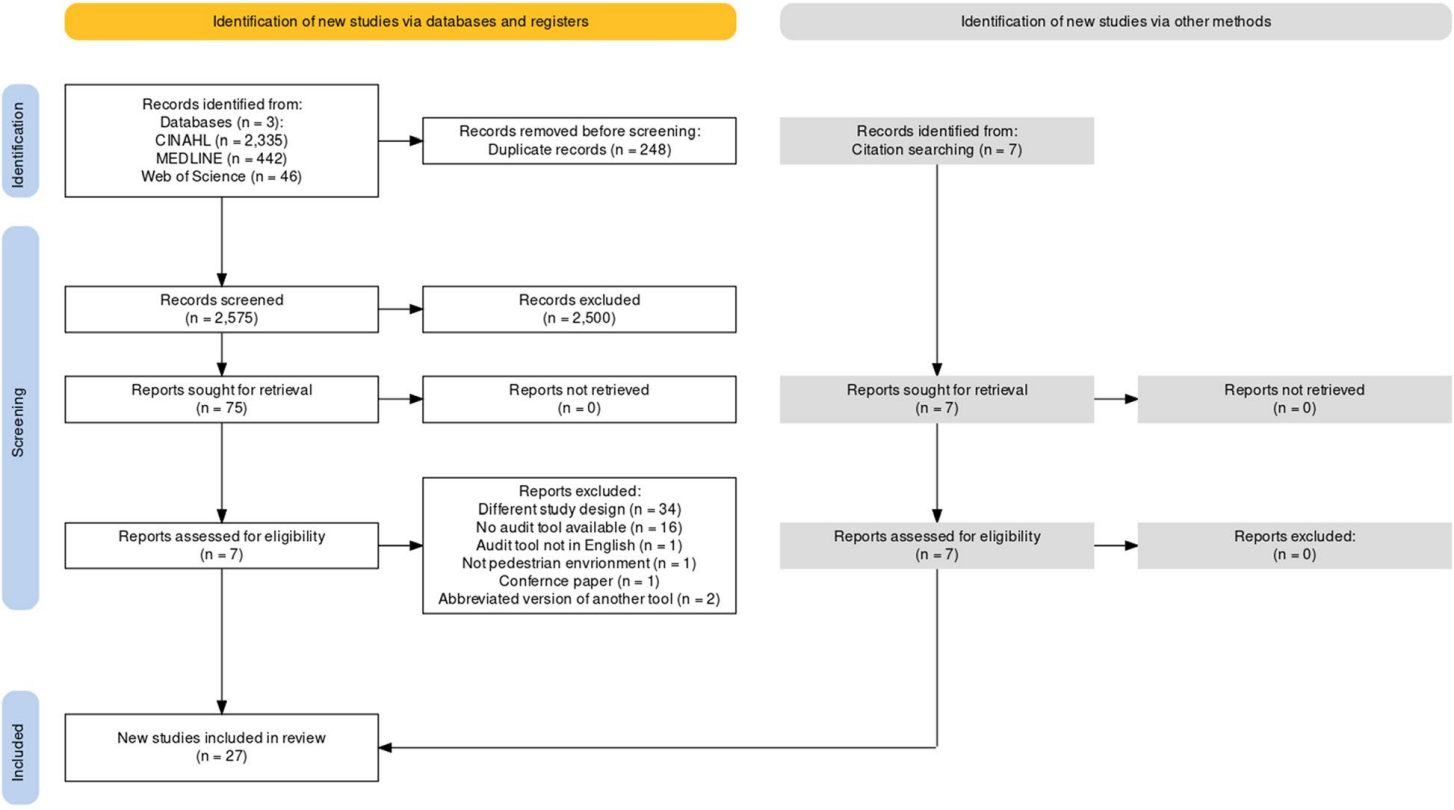
Prisma reporting guidelines for scoping review created using PRISMA2020 [32]

This scoping review was conducted following the framework developed by Arksey and O’Malley [27], the Joanna Briggs Institute (JBI) scoping review guidelines [28], and the JBI systematic review of measurement properties [29]. The protocol can be found in Open Science Framework (OSF) and was publicly released through registration with the OSF platform (https://osf.io/xytwv*)* [30]. To ensure the quality of this review, we referred to the methodological guidelines of the Preferred Reporting Items for Systematic Reviews and Meta-Analysis extension for Scoping Reviews (Fig. 1) [31].

### Search strategy

Search terms and strategy were developed in consultation with a research librarian and in accordance with the PCC (population, concept, context) criteria [33]. Population was determined to be any user of a pedestrian environment and the context was open. Initially, the concept was tools specifically designed for or including weather/season-related built environment features. An initial search was conducted with terms from the concepts, audit tools, pedestrian environment, and meteorological factors. However, no such tools were found in peer-reviewed literature. Thus, the research question was changed to “what are the gaps in the inclusion of weather mitigating built environment features in audit tools, regardless of if they were developed for a specific meteorological condition” from “investigate gaps in the inclusion of weather mitigating built environment in audit tools which consider environmental conditions in their tool”. The context was updated to be any audit tools developed to investigate impacts of the built environment on pedestrian locomotion, and search terms associated with the concept “meteorological factors” were removed from the search. Eligible studies needed to be a methodological paper discussing development, reliability, or validity of a built environment audit tool for pedestrian environments and published in English. Excluded studies did not include built environment elements, were audit tools that were abbreviated versions of prior tools or were of unavailable audit tools.

A literature search was conducted in CINAHL, Medline, and Web of Science. Additional file 1 contains the search strings. Retrieved studies were gathered in RAYYAN online platform to eliminate duplicate and irrelevant references [34]. After this initial process, the remaining publications were transferred to COVIDENCE web-based platform for study screening and data extraction [35].

### Study selection and data extraction

Prior to screening, pilot testing was conducted to ensure high inter-rater agreement between reviewers. Title and abstract pilot screening was conducted by the first reviewer (PS) and a random second reviewer (either AW, FB, or SN). A random sample of 20 studies were screened and PS obtained high agreement with FB (kappa = 1) and SN (kappa = 0.9), and low agreement with AW (kappa = 0.3). Discrepancies between AW and PS were discussed with the reviewing team and with the third reviewer (HD) until consensus was reached. After pilot testing, two random reviewers (either PS, AW, FB, SN) subsequently assessed reference titles and abstracts. Disagreements were resolved with the assistance of an independent third reviewer (HD). Relevant articles were selected for full text screening. At this stage, each full text was evaluated based on the exclusion criteria by two random reviewers (either PS, FB or SN) with conflicts resolved by HD. Finally, included studies were hand searched by PS and any additional studies that met inclusion criteria were included for data extraction.

From the selected studies, data were extracted for psychometric properties of audit tools, including number of built environment items, and reliability and validity testing results. Audit tool characteristics were also extracted including country of development, tool dimensions, and whether the tool was developed specifically for a certain season or weather condition. Next, items included in audit tools were extracted.

To assess the existence of weather/seasonal limitations in audit tools, a list of built environment items that could either mitigate negative effects of weather on pedestrians or promote pedestrian locomotion was compiled after an extensive literature search conducted by the authors. The items were compiled into the following dimensions: temperature (e.g., building material, vegetation), precipitation (e.g., gutters, building overhangs), season (e.g., winter destinations, winter aesthetics), and sustainability (e.g., transit availability).

### Synthesis of findings

Percentages for the inclusion of specific items within each dimension were calculated by dividing the number of audit tools that included a particular item by the total number of audit tools assessed. To compare differences in the inclusion of meteorological items across the included audit tools, capability appraisals for each meteorological dimension were calculated [36] then visualized with a heatmap created through ggplot2 in R (version 4.0.1) [37, 38].

## Results

The initial search returned 2823 documents. After removing duplicates, 2575 documents remained for title and abstract screening; of these, 75 were reviewed in full text. Ultimately, 19 studies from this primary search were included. Citation searches of included studies identified 8 additional documents, which resulted in a total of 27 included studies spanning from 2002 to 2020 (Fig. 1). Though many studies conducted reliability testing on their tool, few (29.63%) discussed the validity.

The length of the items included in the audit tools varied from the smallest number of items included in the sidewalk assessment tool (*n* = 5 items) to large audits that contain up to 191 items (iCHART). There was not a large variation in country of development with most tools (57.69%) being developed in the United States (Table 1). Within the United States, tools were developed across many different regions with varying weather-related concerns. No tool was designed specifically for a certain meteorological condition or season. Furthermore, no audit tool considered meteorological factors specifically to be a dimension.

**Table 1:** General characteristics of included audit tools identified from selected studies

Audit Tool	Country	Reliability Testing	Validity Testing	Included Dimensions
Sidewalk Assessment Tool (SAT) [39]	USA	**✓**	**x**	None
The Pedestrian Environmental Data Scan (PEDS) [40]	USA	**✓**	**x**	(1) Environment (built) (2) Pedestrian Facility (3) Road attributes (4) Walking/cycling environment
St. Louis Analytic Audit Tool (AAT) [41]	USA	**✓**	**x**	(1) Land use (2) Transportation (3) Facilities (4) Aesthetics (5) Signage (6) Social Environment
Systematic Pedestrian and Cycling Environmental Scan (SPACES) [42]	Australia	**✓**	**x**	(1) Walking (2) Streets (3) Safety (4) Permeability (5) Personal Safety (6) Traffic Safety (7) Streetscapes (8) Views (9) Facilities (10) Subjective Assessment
Workplace Walkability Audit Tool (WWAT) [43]	USA	**✓**	**x**	None
Irvine Minnesota Inventory Audit Tool (IMI) [44]	USA	**✓**	**✓**	(1) Accessibility (2) Pleasurably (3) Perceived Safety from Traffic (4) Perceived Safety from Crime
Active Neighbourhood Checklist (ANC) [45]	USA	**✓**	**x**	(1) Land Use Characteristics (2) Sidewalks (3) Street Characteristics (4) Quality of the Environment for Pedestrians
Senior Walking Environmental Assessment Tool (SWEAT-R) [46]	USA	**✓**	**x**	(1) Functionality (2) Aesthetics (3) Safety (4) Destinations
School Environment Audit Tool (TCOPPE) [47]	USA	**✓**	**x**	(1) Land Uses (2) Street and Traffic Characteristics (3) Signage (4) Amenities (5) Social Disorder
Microscale Audit of Pedestrian Streetscapes (MAPS) [48]	USA	**✓**	**x**	(1) Route (Land Use, Streetscape, Aesthetics and Social) (2) Segment (3) Crossing (4) Cul-de-Sac
The Rural Pedestrian Environmental Audit Instrument (REPA) [49]	USA	**✓**	**✓**	(1) Destinations (2) Street Characteristics (3) Aesthetics (4) Social/Dynamic Environment
Wisconsin Assessment of the Social and Built Environment (WASABE) [50]	USA	**✓**	**✓**	1. Neighbourhood Characteristics 2. Transportation Environment 3. Destinations 4. Social Environment 5. Street Connectivity
Madrid Systematic Pedestrian and Cycling Environment Scan (M-SPACES) [51]	Spain	**✓**	**x**	(1) Function (2) Safety (3) Aesthetics (4) Destinations
MAPS global [52]	Belgium	**✓**	**x**	(1) Route (Land Use, Streetscape, Aesthetics and Social) (2) Segment (3) Crossing (4) Cul-de-Sac
Revised Residential Environment Assessment Tool (REAT-2.0) [53]	UK	**✓**	**✓**	(1) Neighbourhood Condition (2) Natural Surveillance (3) Natural Elements (4) Urban Form
Community Health Assessment in Rural Towns (iCHART) [54]	USA	**✓**	**x**	(1) Town Infrastructure (2) Resources 3. Residences
Older People’s Environments and CVD Rick (OPECR) [55]	United Kingdom	**✓**	**✓**	(1) Transport (2) Green Space (3) Advertisements (4) built environment (5) Aesthetics (6) Land Use
Pregnancy, Infection, Nutrition Environmental Audit (PIN3) [56]	USA	**✓**	**x**	(1) Residential Land Use (2) Non-Residential Land Use (3) Public Space (4) Aesthics (5) Mobility Amenities, Transit and Road Characteristics
Computer Assisted Neighbourhood Visual Assessment System (CANVAS) Audit Tool [57]	USA	**✓**	**x**	(1) Building (2) Vegetation and Parks (3) Litter (4) Street conditions (5) Sidewalk Conditions (6) Public Transit
Pittsburgh Hill/Homewood Research on Neighbourhood Change and Health (PHRESH) [58]	USA	**✓**	**x**	(1) Land Use (2) Environment (3) Physical Activity Facility 4. Walking/Cycling Environment 5. Safety Signs 6. Amenities and litter 7. Gathering Places 8. Social Disorder 9. Noise Pollution 10. Physical Disorder
SPOTLIGHT Virtual Audit Tool (S-VAT) [59]	Netherlands	**✓**	**✓**	(1) Walking Related Items (2) Cycling Related Items (3) Public Transport Items (4) Aesthetics Items (5) Land-Use Mix Items (6) Grocery Store Items (7) Food Outlets (8) Recreational Facility Items
Modified S-VAT [60]	Norway	**✓**	**✓**	(1) Walking Related Items (2) Cycling Related Items (3) Public Transport Items (4) Aesthetics Items (5) Land-Use Mix Items (6) Grocery Store Items (7) Food Outlets (8) Recreational Facility Items
School Walkability Index (SWI) [58]	USA	**✓**	**✓**	(1) Land Use Mix (2) Street Characteristics (3) Neighbourhood Perception
FASTVIEW [61]	UK	**✓**	**✓**	(1) Pavement Width and Obstructions (2) Pavement Surface Quality (3) Kerb Paving Quality (4) Road Permeability (5) Way Finding and Legibility (6) Lighting (7) Personal Security (8) User Conflict (9) Environment Quality
China Urban Built Environment Scan Tool (CUBEST) [62]	China	**✓**	**✓**	(1) Residential Density (2) Street Connectivity (3) Accessibility (land-use mix) (4) Sidewalk Quality (5) Bike Lane Quality (6) Aesthetic
Europe Built Environment Outdoor Checklist (CBE-OUT) [63]	Finland, Poland, and Spain	**x**	**x**	1. Streetscape 2. Walkways 3. Bikeways 4. Street Crossing / Intersections 5. Parking Facilities (6) Public Facilities and Features of the Street (7) Land-Use Visible Along the Street / Road (8) Site Decay / Urban Blight (9) Street Activity

### Gaps in meteorological audit tools

**Fig. 2:**
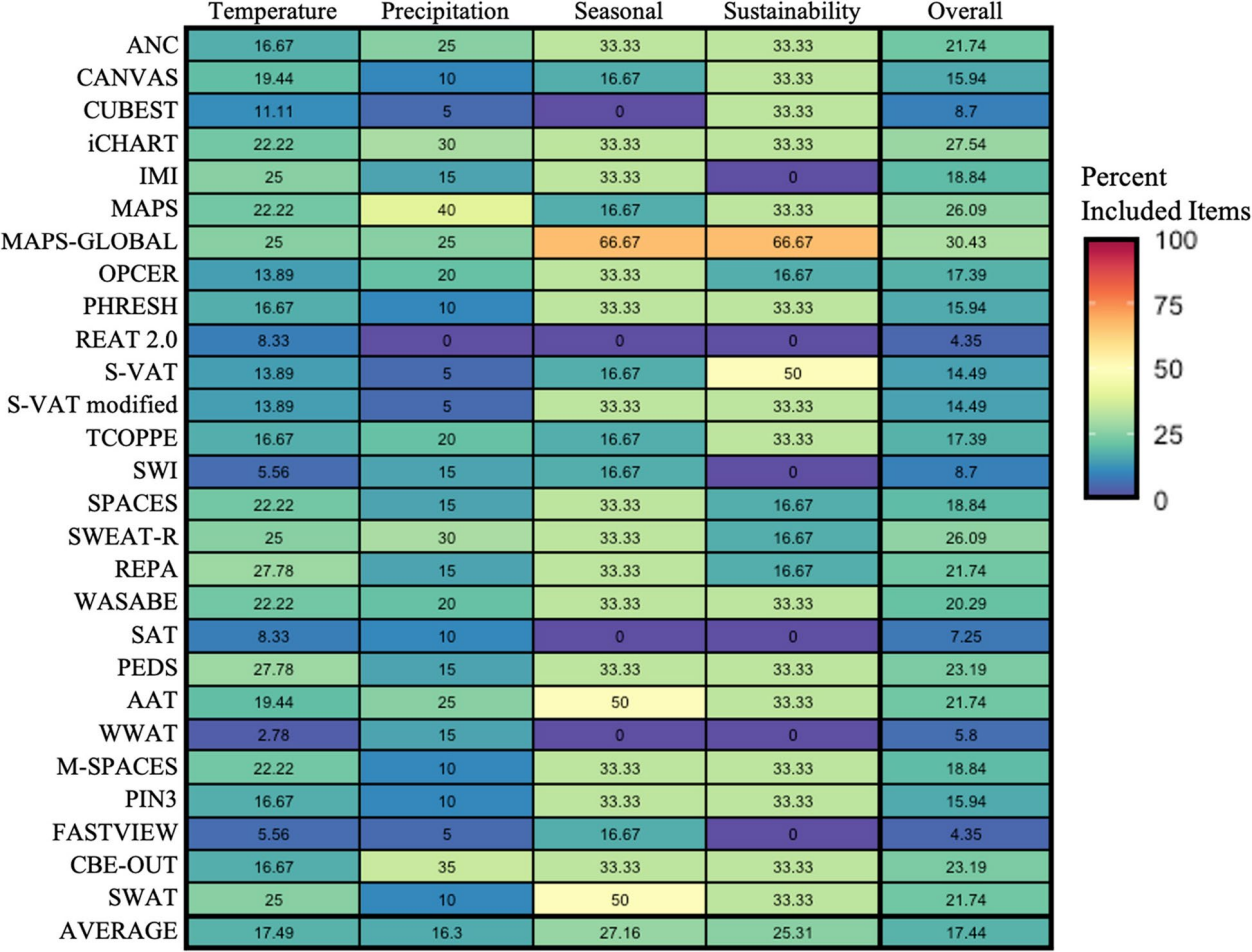
Heatmap showing the percentage of included weather category items in built environment audit tools

 In general, urban design elements to mitigate adverse effects of temperature, precipitation, and seasonal variations, as well as sustainability features to counter future climate change, were not well covered in existing pedestrian environment audits. Most tools included at least one of the items for each category. Every tool included at least one temperature related item, 26 tools included at least one precipitation item, 23 tools included at least one seasonal item, and 21 tools included at least one sustainability item (Tables 2, 3, 4 and 5). Overall, sustainability and seasonal categories exhibited the most extensive coverage, with an average inclusion rate across audit tools of 27.16% and 25.31%, respectively (Fig. 2). Conversely, temperature and precipitation categories had the lowest coverage, averaging 17.49% and 16.3%, respectively, across audit tools (Fig. 2). Among the audit tools assessed, the Microscale Audit of Pedestrian Streetscapes (MAPS) global contained the highest number of items, encompassing 30.43% of the possible 69 meteorological items, while the Revised Residential Environment Assessment Tool (REAT 2.0) had the lowest number of items, covering just 4.35% of the potential items (Fig. 2).

**Table 2:** Inclusion of environmental features that might alter the thermal comfort of pedestrians in audit tools developed to assess pedestrian environments

**Feature**	**ANC**	**CANVAS**	**CUBEST**	**iCHART**	**IMI**	**MAPS**	**MAPS-GLOBAL**	**OPCER**	**PHRESH**	**REAT 2.0**	**S-VAT**	**SVAT-modified**	**TCOPPPE**	**SWI**
Indoor public space [19, 20]	**✓**		**✓**	**✓**	**✓**	**✓**	**✓**	**✓**	**✓**		**✓**	**✓**	**✓**	
Direct Cooling [64, 65]							**✓**							
Direct Heating [19]					**✓**									
Arctic entry / vestibule [19]														
Building height [19, 66]				**✓**	**✓**	**✓**	**✓**							
Building material [18, 21, 67]														
Sidewalk material [18, 20, 21, 67, 68]		**✓**		**✓**									**✓**	
Road material [18, 21, 63, 67]														
Roof material [18, 21, 67]														
Furniture material [19, 69, 70]														
Building colour [18, 19, 21, 67]														
Sidewalk colour [18, 21, 67, 71]														
Road colour [18, 21, 67, 71]														
Roof colour [18, 21, 67, 71]														
Furniture colour [18, 21, 67, 71]														
Building texture [18, 21, 67]														
Sidewalk texture [18, 21, 67]														
Road texture [18, 21, 67]														
Roof texture [18, 21, 67]														
Green roofs^a^ [18, 20, 21, 72]														
Green walls [18, 21]														
Blue roofs [18, 21]														
Blue walls [18, 21]														
Wind barriers [19]														
Building setback [73]				**✓**		**✓**	**✓**							
Building step back [19]														
Shade structures [18]														
Shade coverage^a^ [21, 70, 71]	**✓**			**✓**	**✓**	**✓**	**✓**		**✓**				**✓**	
Presence of trees^a^ [19, 21, 74]	**✓**	**✓**		**✓**	**✓**	**✓**	**✓**	**✓**	**✓**	**✓**	**✓**	**✓**	**✓**	**✓**
Tree species^a^ [19, 21]														
Presence of shrubs^a^ [74]		**✓**						**✓**						
Presence of grass^a^ [71]	**✓**	**✓**		**✓**				**✓**						
Presence of maintained green spaces^a^ [21]	**✓**	**✓**	**✓**	**✓**	**✓**	**✓**	**✓**	**✓**	**✓**	**✓**	**✓**	**✓**	**✓**	**✓**
Presence of natural green space^a^ [21]	**✓**	**✓**		**✓**	**✓**				**✓**		**✓**	**✓**		
Presence of maintained blue space [21, 75]														
Presence of natural blue space [21, 74, 75]			**✓**		**✓**	**✓**	**✓**			**✓**	**✓**	**✓**		

**Feature**	**SPACES**	**SWEAT-*R***	**RPEA**	**WASABE**	**SAT**	**PEDS**	**AAT**	**WWAT**	**M-SPACES**	**PIN3**	**FASTVIEW**	**CBE-OUT**	**SWAT**	**Item Coverage (%)**
Indoor public space [19, 20]	**✓**	**✓**	**✓**	**✓**		**✓**	**✓**		**✓**			**✓**	**✓**	74
Direct Cooling [64, 65]														4
Direct Heating [19]														4
Arctic entry / vestibule [19]														0
Building height [19, 66]		**✓**				**✓**								22
Building material [18, 21, 67]						**✓**								4
Sidewalk material [18, 20, 21, 67, 68]	**✓**	**✓**	**✓**			**✓**			**✓**				**✓**	33
Road material [18, 21, 63, 67]		**✓**												4
Roof material [18, 21, 67]														0
Furniture material [19, 69, 70]														0
Building colour [18, 19, 21, 67]														0
Sidewalk colour [18, 21, 67, 71]														0
Road colour [18, 21, 67, 71]														0
Roof colour [18, 21, 67, 71]														0
Furniture colour [18, 21, 67, 71]														0
Building texture [18, 21, 67]														0
Sidewalk texture [18, 21, 67]														0
Road texture [18, 21, 67]														0
Roof texture [18, 21, 67]														0
Green roofs^a^ [18, 20, 21, 72]														0
Green walls [18, 21]														0
Blue roofs [18, 21]														0
Blue walls [18, 21]														0
Wind barriers [19]														0
Building setback [73]						**✓**								15
Building step back [19]														0
Shade structures [18]														0
Shade coverage^a^ [21, 70, 71]			**✓**			**✓**		**✓**		**✓**				41
Presence of trees^a^ [19, 21, 74]	**✓**	**✓**	**✓**	**✓**	**✓**	**✓**	**✓**		**✓**	**✓**	**✓**	**✓**	**✓**	93
Tree species^a^ [19, 21]														0
Presence of shrubs^a^ [74]			**✓**	**✓**	**✓**	**✓**				**✓**			**✓**	30
Presence of grass^a^ [71]	**✓**	**✓**	**✓**	**✓**	**✓**	**✓**			**✓**	**✓**			**✓**	48
Presence of maintained green spaces^a^ [21]	**✓**	**✓**	**✓**	**✓**			**✓**		**✓**	**✓**		**✓**	**✓**	85
Presence of natural green space^a^ [21]	**✓**	**✓**	**✓**						**✓**			**✓**	**✓**	48
Presence of maintained blue space [21, 75]				**✓**			**✓**						**✓**	11
Presence of natural blue space [21, 74, 75]	**✓**	**✓**	**✓**				**✓**		**✓**			**✓**		48

^a^Item is also important for mitigating the impact of precipitation

**Table 3:** Inclusion of environmental features that may mitigate the negative impact of precipitation on pedestrians in audit tools developed to assess pedestrian environments

**Feature**	**ANC**	**CANVAS**	**CUBEST**	**iCHART**	**IMI**	**MAPS**	**MAPS-GLOBAL**	**OPCER**	**PHRESH**	**REAT 2.0**	**S-VAT**	**SVAT-modified**	**TCOPPPE**	**SWI**
Building overhangs [19, 20, 69]				**✓**	**✓**	**✓**	**✓**						**✓**	
Covered walkways [19, 20, 69]		**✓**		**✓**	**✓**	**✓**	**✓**	**✓**						
Covered transit station [19]				**✓**		**✓**	**✓**	**✓**	**✓**					
Pedways [19, 69]														
Presence of puddles [19, 20]														**✓**
Presence of snow on sidewalk [19, 76]														
Presence of ice on sidewalk [19, 76]														
Gutters [77]	**✓**			**✓**		**✓**								
Permeable pavements [72]														
Bioswales [72]														
Rain gardens [72]														
Storm drains / catch basins [19, 77]	**✓**													
Drainage ditches [72]					**✓**	**✓**							**✓**	
Buffer zone [19, 20, 72]	**✓**	**✓**		**✓**		**✓**	**✓**	**✓**	**✓**		**✓**	**✓**	**✓**	**✓**
Roof slope [19]														
Heated sidewalk [19, 69]														
Parking ban [19]														
Sidewalk width [19]	**✓**		**✓**	**✓**		**✓**		**✓**					**✓**	**✓**
Bike lane width [19]	**✓**													
Aligned curb cuts [19]						**✓**	**✓**							
Handrails [19, 69]														

**Feature**	**SPACES**	**SWEAT-*R***	**RPEA**	**WASABE**	**SAT**	**PEDS**	**AAT**	**WWAT**	**M-SPACES**	**PIN3**	**FASTVIEW**	**CBE-OUT**	**SWAT**	**Item Coverage (%)**
Building overhangs [19, 20, 69]		**✓**												22
Covered walkways [19, 20, 69]		**✓**										**✓**		30
Covered transit station [19]		**✓**				**✓**	**✓**					**✓**		33
Pedways [19, 69]														0
Presence of puddles [19, 20]								**✓**				**✓**		11
Presence of snow on sidewalk [19, 76]				**✓**	**✓**							**✓**		11
Presence of ice on sidewalk [19, 76]				**✓**	**✓**							**✓**		11
Gutters [77]														11
Permeable pavements [72]														0
Bioswales [72]														0
Rain gardens [72]														0
Storm drains / catch basins [19, 77]				**✓**			**✓**					**✓**		15
Drainage ditches [72]			**✓**											15
Buffer zone [19, 20, 72]	**✓**	**✓**	**✓**	**✓**		**✓**	**✓**	**✓**	**✓**	**✓**		**✓**	**✓**	81
Roof slope [19]														0
Heated sidewalk [19, 69]														0
Parking ban [19]	**✓**								**✓**					7
Sidewalk width [19]	**✓**	**✓**	**✓**			**✓**	**✓**	**✓**		**✓**	**✓**		**✓**	59
Bike lane width [19]							**✓**							7
Aligned curb cuts [19]		**✓**												11
Handrails [19, 69]														0

**Table 4:** Inclusion of seasonal items that might impact pedestrian PA in audit tools developed to assess pedestrian environments

**Feature**	**ANC**	**CANVAS**	**CUBEST**	**iCHART**	**IMI**	**MAPS**	**MAPS-GLOBAL**	**OPCER**	**PHRESH**	**REAT 2.0**	**S-VAT**	**SVAT-modified**	**TCOPPPE**	**SWI**
Summer destinations [70, 78–80]	**✓**			**✓**	**✓**		**✓**	**✓**	**✓**			**✓**		
Winter destination [19, 69, 78]														
Winter aesthetics [19, 69, 81]														
Seasonal amenities [19, 69, 70]							**✓**							
Lighting [19, 20, 69]		**✓**		**✓**	**✓**	**✓**	**✓**	**✓**	**✓**		**✓**	**✓**	**✓**	**✓**
Pedestrian scale lighting [19, 20, 69]	**✓**						**✓**							

**Feature**	**SPACES**	**SWEAT-*R***	**RPEA**	**WASABE**	**SAT**	**PEDS**	**AAT**	**WWAT**	**M-SPACES**	**PIN3**	**FASTVIEW**	**CBE-OUT**	**SWAT**	**Item Coverage (%)**
Summer destinations [70, 78–80]		**✓**	**✓**	**✓**			**✓**					**✓**	**✓**	48
Winter destination [19, 69, 78]														0
Winter aesthetics [19, 69, 81]														0
Seasonal amenities [19, 69, 70]	**✓**						**✓**		**✓**				**✓**	19
Lighting [19, 20, 69]	**✓**	**✓**	**✓**	**✓**		**✓**	**✓**		**✓**	**✓**	**✓**	**✓**	**✓**	81
Pedestrian scale lighting [19, 20, 69]						**✓**				**✓**				15

**Table 5:** Inclusion of sustainability items that might impact pedestrian PA in audit tools developed to assess pedestrian environments

**Feature**	**ANC**	**CANVAS**	**CUBEST**	**iCHART**	**IMI**	**MAPS**	**MAPS-GLOBAL**	**OPCER**	**PHRESH**	**REAT 2.0**	**S-VAT**	**SVAT-modified**	**TCOPPPE**	**SWI**
Renewable energy [82]														
Transit access [70, 82]	**✓**	**✓**	**✓**	**✓**		**✓**	**✓**	**✓**	**✓**		**✓**	**✓**	**✓**	
Bike lanes [70, 82]	**✓**	**✓**	**✓**	**✓**		**✓**	**✓**		**✓**		**✓**	**✓**	**✓**	
Bike / scoter share facilities [70]							**✓**				**✓**			
Car share [70]							**✓**							
Electric vehicle charging [70]														

**Feature**	**SPACES**	**SWEAT-*R***	**RPEA**	**WASABE**	**SAT**	**PEDS**	**AAT**	**WWAT**	**M-SPACES**	**PIN3**	**FASTVIEW**	**CBE-OUT**	**SWAT**	**Item Coverage (%)**
Renewable energy [82]														0
Transit access [70, 82]		**✓**	**✓**	**✓**		**✓**	**✓**		**✓**	**✓**		**✓**	**✓**	74
Bike lanes [70, 82]	**✓**			**✓**		**✓**	**✓**		**✓**	**✓**		**✓**	**✓**	67
Bike / scoter share facilities [70]														7
Car share [70]														4
Electric vehicle charging [70]														0

Pedestrian environment audit tools included 2.78% (Workplace Walkability Audit Tool, WWAT) – 27.78% (Pedestrian Environmental Data Scan, PEDS) of temperature-related items (Fig. 2). Certain items had high coverage within tools such as “indoor public spaces” (74%), “the presence of trees” (93%), and the “existence of maintained green spaces” (85%), all of which were featured in nearly all audit tools (Table 2). Items such as shade coverage (41%), the presence of natural green spaces (48%), natural blue spaces (48%), grass (48%), shrubs (30%), building height (22%), and sidewalk material (33%) received moderate coverage (Table 2). Certain items were rarely covered, with only MAPS global including them, such as direct cooling, direct heating, and roadway material. Moreover, several temperature-related items were completely absent from all audit tools, including the presence of an arctic entry/vestibule, furniture material, colours of sidewalks, roads, buildings, and furniture, textures of sidewalks, roads, buildings, and furniture, as well as the presence of blue or green roofs or walls and built shade structures (Table 2).

Precipitation was the least covered category by audit tools with an average coverage of 16% (Fig. 2), most covered by MAPS (40%) and least covered by REAT 2.0 (0%) (Fig. 2). The most included item was the presence of a buffer zone, which was included in 81% of audits. Moderately covered items included sidewalk width (59%), covered walkways (30%), presence of a transit shelter (33%), and building overhangs (22%) (Table 3). In some audit tools, the items puddle presence (11%), snow maintenance (11%), ice maintenance (11%), gutter presence (11%), drain presence (15%), drainage ditch presence (15%), parking ban (7%), bike lane width (7%), and aligned curb cuts (11%) were included (Table 3).

Seasonal items were more frequently covered than temperature or precipitation items with 27% of items being included in audit tools on average (Fig. 2). The tool that included the most seasonal items was the MAPS-Global, which included four seasonal items (summer destinations, seasonal amenities, lighting, and pedestrian scale lighting) (Fig. 2; Table 4). The audits that contained the least number of items were the WWAT, Sidewalk Assessment Tool (SAT), REAT 2.0, and the China Urban Built Environment Scan Tool (CUBEST) (Fig. 2). The highest covered item was the presence of lighting, which was included in 81% of tools, followed by the inclusion of summer destinations which was included in 48% of tools. The remaining items were much less considered. Seasonal amenities (19%) and pedestrian scale lighting (15%) were considered in some audits, while winter destinations and aesthetics were never included (Table 4).

On average across tools, sustainability items had 25% coverage (Fig. 2). MAPS-Global included the most features including all items except for “electric vehicle charging stations” and “renewable energy”, both of which were not covered by any tool (Fig. 2; Table 5). Tools with the lowest coverage in this category were REAT 2.0, School Walkability Index (SWI), SAT, and WWAT, which did not include any of the possible sustainability items (Fig. 2). The remaining tools covered at least one to three sustainability items. The most well-covered sustainability items included transit access (74%) and bike lanes (67%), whereas car or bike share facilities were rarely included (4% and 7%, respectively) (Table 5).

## Discussion

This scoping review had two primary aims: (1) to investigate whether any built environment audit tools were specifically developed for use during a specific season or weather condition, and (2) to investigate gaps in the inclusion of weather mitigating items within built environment audit tools, regardless of whether they were developed for a specific meteorological condition. No peer-reviewed pedestrian environment audit tools developed for use during specific weather conditions or seasons were identified. Moreover, and consistent with previous findings [83], no existing tool considered weather or season as dimensions within their audit (Table 1). Despite the absence of peer-reviewed audit tools, a non-peer-reviewed community-based audit tool called the Snow Mole audit has been developed by the Council on Aging of Ottawa [84]. It is a volunteer-driven initiative aimed at assessing the safety of Ottawa’s sidewalks during winter and includes nine dichotomous items, such as the presence of ice and snow on sidewalks, handrails, and snowbanks [84]. While this questionnaire represents progress in measuring the accessibility of winter pedestrian environments, it lacks items related to winter destinations (e.g., skating rinks, ski hills), aesthetics (e.g., ice castles), or heating features (e.g., fireplaces, shelters) that may also be essential for encouraging pedestrian use [85]. Additionally, the checklist only assesses the presence or absence of winter features, rather than their extent. This limitation is significant since both researchers and built environment policy advocates require a detailed, systematic, reliable, and valid audit tool [48].

### Inclusion of features for meteorological mitigation associated with other domains

General built environment audit tools lack the capability to measure the impact of environmental features that could mitigate the negative effects of weather and seasons on pedestrians, with most tools (92.6%) capturing less than 33% of items in any dimension (temperature, precipitation, season, or sustainability). However, certain indicators are well-incorporated within audit tools, such as the presence of trees, lighting, and indoor public spaces. Other indicators, such as winter destinations and building colours, are rarely, if ever, considered (Tables 2, 3, 4 and 5).

One explanation for the high coverage of certain meteorological indicators may be their association with other physical activity domains. For instance, indoor public spaces typically fall under the land use dimension, which is associated with increased active transport [83, 86, 87]. Similarly, the presence of maintained green spaces falls under the dimension of access to recreational facilities and is associated with lower risk for obesity [83, 88]. Lighting is associated with safety dimensions as it is found to reduce crime, therefore indirectly increasing walking behavior, albeit with mixed evidence [83, 89]. Finally, the presence of a buffer zone typically falls under sidewalk or safety, providing a barrier separating pedestrians from cars [83, 90]. An exception is the well-covered item “presence of trees,” frequently used to assess the level of shade on sidewalks (thereby reducing temperature). The high coverage of certain meteorological mitigation items that overlap with other audit domains may indicate their inclusion is likely not due to meteorological reasons but, rather, with their association in other domains. Further research could explore assumed meanings by auditors using each tool to ensure valid interpretation of survey items.

### Gaps in features for thermal comfort

Features of the built environment can significantly influence the local microclimate and amount of solar radiation an area receives which can either increase or decrease thermal comfort [16, 18, 91]. Vegetation, for instance, plays a substantial role in reducing microclimate temperatures through evapotranspiration [21, 71, 74]. Building morphology can also reduce microclimate temperatures through the creation of wind tunnel [92]. Morphology can also impact the amount of solar radiation in a given area, for example, high density streets with buildings that have a high height to width ratio decrease the portion of visible sky above an area (sky view factor) thus decreasing the solar radiation of an area which can increase thermal comfort [91]. Additionally, albedo (a product of material, colour, and texture) or built structures can alter street temperatures [18, 21, 67, 71]. Objects with lower albedo tend to absorb more solar radiation, while those with high albedo reflect a significant amount of radiation; therefore, designing streets with high albedo objects can result in lower temperatures compared to streets designed with low albedo structures. Although the presence of green spaces and vegetation is moderately covered in built environment assessments, this focus is likely due to their association with various walkability aspects such as aesthetics. Surprisingly, these assessments seldom include considerations of built features that can directly influence street temperatures and solar radiation.

Similarly, features intentionally designed to reduce street temperatures, such as blue and green roofs and walls, which operate by promoting cooling through evaporation, are almost never incorporated in audit tools. Beyond passive mechanisms to alter temperature, an alternate approach is to include features that directly heat or cool pedestrian spaces [19, 64, 65]. Again, these features were rarely considered. This observation provides further evidence that current audit tools are not considering weather impacts in their audits and highlights a noticeable gap: failure to account for features that could directly impact the temperature of street environments. This is an important feature to consider since PA decreases considerably during times of high temperatures [7].

### Gaps in features to protect against precipitation

To protect pedestrians from the negative impacts of precipitation, pedestrians need protection from overhead rain or snow, and the presence of water, snow, or ice on sidewalks [20, 93]. While there is moderate coverage of items in the audit tools that can protect pedestrians from overhead rain or snow, there is limited consideration of the impact of precipitation on sidewalk maintenance or the management of these hazards. Precipitation hazards can be prevented through urban design features, such as runoff management features or, in the case of ice and snow, can be managed through city snow clearing policies [72, 93]. For rain (and snowmelt), preventive measures can include features that channel water away from the sidewalk (e.g., gutters, drains, bioswales) or permeable pavements, allowing precipitation to filter through the pavement and into a drainage system below [72]. The buildup of snow and ice can be prevented with heated sidewalks to melt the snow, or managed with snow removal aided by features such as sidewalk width and aligned curb cuts [19, 94]. Along with limited inclusion of prevention/management of hazards, audits also rarely include items assessing presence of precipitation hazards on sidewalks. These limitations together represent a significant gap in audit tools as individuals with reduced mobility can face substantial barriers to PA due to presence of ice, snow, or puddles on sidewalks [93].

### Gaps in winter features

PA is often at its lowest during winter months [2, 95]; therefore, it is especially important for cities in cold regions to employ design features to prevent this drop in activity. The winter city movement is aimed at increasing livability in winter cities, primarily through increased use of outdoor public spaces by inhabitants [96]. Proponents recommend features such as increased lighting, winter-specific aesthetics (e.g., snow art), and destinations to increase use of these spaces [69, 96]. Interventions where winter destinations and aesthetic areas were implemented have found increases in pedestrian engagement with the built environment [78]. The lack of inclusion of winter destinations, aesthetics, and lighting paired with limited inclusion of precipitation and temperature features all point to inability in current audit tools to measure winter pedestrian spaces.

### Recommendations

Given the substantial impact of weather and season on PA [2–6], the lack of inclusion of features that could mitigate this in existing audit tools is a substantial gap. This issue may be less critical in regions with generally mild temperatures throughout the year and brief precipitation events. However, in areas with extended winter seasons, extreme heat, or prolonged periods of rain, this becomes a significant issue. Therefore, it is imperative to develop built environment audit tools to measure presence and extent of features that can mitigate weather’s negative impacts on pedestrians. This review specifically identifies the need for a winter-specific audit tool for cold regions, a rain audit tool for rainy regions, and general inclusion of built features that alter microclimates in audits, as areas worldwide experience continued climate change.

### Limitations

This study has several limitations. First, the list of meteorological indicators is by no means exhaustive and was compiled by the authors based on a preliminary scan of the literature; therefore, it may be lacking in the inclusion of urban design features that are important in mitigating the impact of weather or season on pedestrians. Second, grey literature was not included in the search, and this review may not provide a complete picture of meteorological mitigating features in audit tools.

## Conclusions

This study identified significant gaps in existing built environment audit tools, none of which were specifically designed to address the impacts of different weather conditions and seasons. These gaps, which include inadequate measures for temperature regulation, precipitation management, seasonal features, and sustainability, represent a critical need for tools that more comprehensively assess how urban design influences pedestrian activity and comfort.

Future research should focus on developing such comprehensive or complementary audit tools that integrate weather and season-specific features. These tools should incorporate assessments for temperature regulation, such as green roofs, shaded walkways, and materials with high albedo; effective precipitation management such as advanced drainage systems and permeable pavements; enhanced seasonal usability of amenities designed for winter and summer use; and sustainability measures like renewable energy sources, electric vehicle charging stations, and sustainable transportation.

By addressing these areas, future research can be used to develop tools to improve the comprehensiveness of assessments of pedestrian environments that fully address today’s health and climate priorities. Such assessments in turn can be used to foster urban space improvements to promote physical activity, and resilience against climate change. This review marks an important step in identifying the meteorological gaps in current built environment audit tools, demonstrating needed advancements to fully address our environments for human health and environmental sustainability.

### Supplementary Information

Supplementary Material 1.

## Data Availability

No datasets were generated or analysed during the current study.

## Abbreviations

PA: Physical Activity
SAT: Sidewalk Assessment Tool
PEDS: Pedestrian Environmental Data Scan
AAT: St. Louis Analytic Audit Tool
SPACES: Systematic Pedestrian and Cycling Environmental Scan
WWAT: Workplace Walkability Audit Tool
IMI: Irvine Minnesota Inventory
ANC: Active Neighbourhood Checklist
SWEAT-R: Revised Senior Walking Environmental Assessment Tool
TCOPPE: School Environment Audit Tool
MAPS: Microscale Audit of Pedestrian Streetscapes
REPA: The Rural Pedestrian Environmental Audit Instrument
WASABE: Wisconsin Assessment of the Social and Built Environment
M-SPACES: Madrid Systematic Pedestrian and Cycling Environment Scan
REAT-2.0: Revised Residential Environment Assessment Tool
iCHART: Community Health Assessment in Rural Towns
OPECR: Older People's Environments and CVD Rick
PIN3: Pregnancy, Infection, Nutrition Environnemental Audit
CANVAS: Computer Assisted Neighbourhood Visual Assessment System
PRESH: Pittsburgh Hill/Homewood Research on Neighbourhood Change and Health
S-VAT: SPOTLIGHT Virtual Audit Tool
CUBEST: China Urban Built Environment Scan Tool
SWI: School Walkability Index
