# Supplementary material for: Meteorological gaps in audits of pedestrian environments: a scoping review
Source: BMC Public Health. 2024 Jul 27;24:2010. doi: 10.1186/s12889-024-19441-6 (PMC11282675; doi:10.1186/s12889-024-19441-6)
Supplement: Supplementary file 1 — Supplementary Material 1. [file 12889_2024_19441_MOESM1_ESM.docx]

**Table S1:** Search string utilized when executing database searches. Searches were executed from the date of the databases inception to June 13, 2023.

| **Database** | **String** |
| --- | --- |
| MEDLINE | ("evaluation tool*" or "assessment tool*" or "measurement instrument*" or "audit tool*" or "audit instrument*").mp. [mp=title, book title, abstract, original title, name of substance word, subject heading word, floating sub-heading word, keyword heading word, organism supplementary concept word, protocol supplementary concept word, rare disease supplementary concept word, unique identifier, synonyms, population supplementary concept word, anatomy supplementary concept word] AND (pedestrian* or "active transport*" or bicycl* or cyclist* or cycling or "rollerblading" or wheelchair* or sidewalk* or walkab* or "built environment*" or "urban design" or "urban planning" or "active commuting" or neighborhood* or "street design").mp. [mp=title, book title, abstract, original title, name of substance word, subject heading word, floating sub-heading word, keyword heading word, organism supplementary concept word, protocol supplementary concept word, rare disease supplementary concept word, unique identifier, synonyms, population supplementary concept word, anatomy supplementary concept word] |
| Web of Science | "evaluation tool*" or "assessment tool*" or "measurement instrument*" or "audit tool*" or "audit instrument*" (Topic) and "Central corridor pedestrian environment" or "Systematic pedestrian and cycling environmental scan" or "Pedestrian environment data scan" or "active neighborhood checklist" or "analytic audit tool" or "Irvine Minnesota inventory" or "walking suitability assessment form" or "neighborhood audit tool" or "path environment audit tool" (All Fields) and pedestrian* or "active transport*" or bicycl* or cyclist* or cycling or "rollerblading" or wheelchair* or sidewalk* or walkab* or "built environment*" or "urban design" or "urban planning" or "active commuting" or neighborhood* or "street design" (All Fields) |
| CINHAL | ( "evaluation tool*" or "assessment tool*" or "measurement instrument*" or "audit tool*" or "audit instrument*" ) AND ( pedestrian* or "active transport*" or bicycl* or cyclist* or cycling or "rollerblading" or wheelchair* or sidewalk* or walkab* or "built environment*" or "urban design" or "urban planning" or "active commuting" or neighborhood* or "street design" ) |
